# Changes in left ventricular electromechanical relations during targeted hypothermia

**DOI:** 10.1186/s40635-020-00363-7

**Published:** 2020-12-14

**Authors:** Kristin Wisløff-Aase, Viesturs Kerans, Kristina Haugaa, Per Steinar Halvorsen, Helge Skulstad, Andreas Espinoza

**Affiliations:** 1 Department of Anaesthesiology, Oslo University Hospital – Rikshospitalet, Nydalen, PO Box 4950, 0424 Oslo, Norway; 2 Institute of Clinical Medicine, Faculty of Medicine, University of Oslo, Oslo, Norway; 3 Department of Cardiology, Oslo University Hospital, Oslo, Norway; 4 The Intervention Centre, Oslo University Hospital, Oslo, Norway

**Keywords:** Targeted hypothermia, Ventricular arrhythmia, Echocardiography, Myocardial function, Electromechanical relations, Electromechanical window

## Abstract

**Background:**

Targeted hypothermia, as used after cardiac arrest, increases electrical and mechanical systolic duration. Differences in duration of electrical and mechanical systole are correlated to ventricular arrhythmias. The electromechanical window (EMW) becomes negative when the electrical systole outlasts the mechanical systole. Prolonged electrical systole corresponds to prolonged QT interval, and is associated with increased dispersion of repolarization and mechanical dispersion. These three factors predispose for arrhythmias. The electromechanical relations during targeted hypothermia are unknown.

We wanted to explore the electromechanical relations during hypothermia at 33 °C. We hypothesized that targeted hypothermia would increase electrical and mechanical systolic duration without more profound EMW negativity, nor an increase in dispersion of repolarization and mechanical dispersion.

**Methods:**

In a porcine model (*n* = 14), we registered electrocardiogram (ECG) and echocardiographic recordings during 38 °C and 33 °C, at spontaneous and atrial paced heart rate 100 beats/min. EMW was calculated by subtracting electrical systole; QT interval, from the corresponding mechanical systole; QRS onset to aortic valve closure. Dispersion of repolarization was measured as time from peak to end of the ECG T wave. Mechanical dispersion was calculated by strain echocardiography as standard deviation of time to peak strain.

**Results:**

Electrical systole increased during hypothermia at spontaneous heart rate (*p* < 0.001) and heart rate 100 beats/min (*p* = 0.005). Mechanical systolic duration was prolonged and outlasted electrical systole independently of heart rate (*p* < 0.001). EMW changed from negative to positive value (− 20 ± 19 to 27 ± 34 ms, *p* = 0.001). The positivity was even more pronounced at heart rate 100 beats/min (− 25 ± 26 to 41 ± 18 ms, *p* < 0.001). Dispersion of repolarization decreased (*p* = 0.027 and *p* = 0.003), while mechanical dispersion did not differ (*p* = 0.078 and *p* = 0.297).

**Conclusion:**

Targeted hypothermia increased electrical and mechanical systolic duration, the electromechanical window became positive, dispersion of repolarization was slightly reduced and mechanical dispersion was unchanged. These alterations may have clinical importance. Further clinical studies are required to clarify whether corresponding electromechanical alterations are accommodating in humans.

## Background

Targeted hypothermia (32–36 °C) is recommended in comatose cardiac arrest survivors to improve outcome [1]. However, hypothermia alters cardiac electrical and mechanical function, with reduced heart rate, increased QT interval on the electrocardiogram (ECG) and prolonged mechanical systolic duration [2, 3]. Electrical and mechanical myocardial physiology is closely coupled and under normal circumstances mechanical systole terminates just milliseconds after electrical systole (QT interval) giving a positive electromechanical window (EMW). In certain conditions where the QT interval, outlasts the mechanical systole, this leads to negative EMW. QT interval prolongation is associated with dispersion of repolarization and mechanical dispersion. An increased QT interval, [4, 5] electromechanical window negativity, dispersion of repolarization and mechanical dispersion are correlated to arrhythmic events under a wide diversity of clinical conditions [6–10].

However, there has not been observed increased incidence of adverse ventricular arrhythmic events during treatment with targeted hypothermia in cardiac arrest survivors [11–13]. EMW, dispersion of repolarization and mechanical dispersion are all novel parameters with clinical relevance to understand the effect of hypothermia and are not previously analysed and described. The present experimental animal study aimed to explore the electromechanical relations during targeted hypothermia at 33 °C. We hypothesized that hypothermia increases electrical and mechanical systolic duration without more profound EMW negativity independently of heart rate. Furthermore, we hypothesized that dispersion of repolarization and mechanical dispersion, remain unchanged during hypothermia.

## Methods

### Animal model

The experiment was performed in an open chest porcine model. Data for the present study were collected from experiment series published in two previous articles [3, 14]. The open model facilitated recording of high quality echocardiographic images needed for the study, otherwise difficult to obtain in the pigs.

The study was approved by Nationals Animal Research Authority of Norway (trial registration number: FOTS 3866) and carried out in accordance to the European Convention for the protection of vertebrate animals used for experimental and other scientific purposes, the European Union Directive 2010/63/EU [15].

### Animal preparations

The animals (*n* = 14, Norwegian land race swine, mean weight 52 ± 4.3 kg) were fasting overnight aside from free water access. They were pre-medicated by an intramuscular injection with ketamine (20 mg/kg), azaperone (3 mg/kg) and atropine (20 µg/kg). Anaesthesia was induced with intravenous pentobarbital (3 mg/kg) and morphine (2 mg/kg), and maintained with morphine infusion (1–2 mg/kg/h) and isoflurane inhalation (1–1.5%). Neuromuscular blocking agents were not used. Level of anaesthesia was monitored by continually haemodynamic measurements and observation of clinical signs. When the protocol was finished, the animals were euthanatized by bolus infusion of 80 mmol potassium chloride and 1000 mg pentobarbital. The animals were mechanically ventilated via a tracheostomy tube by a fraction of air/oxygen (FiO_2_ 0.4), with tidal volumes of 10–15 ml/kg and surgically prepared with sternotomy as previously reported [3, 14]. Three-lead ECG was obtained by skin leads. Pacemaker leads were sutured to the right atrium. For temperature control, a water circulated catheter (Cool Line; Zoll, Chelmsford, MA, USA) was inserted into the inferior vena cava by cannulation of the left femoral vein and connected to the thermal regulation system (Coolgard 3000; Zoll). A pulmonary artery catheter (Swan-Ganz CCO; Edwards Lifesciences, Irvin, CA, USA) was inserted through the right jugular vein to measure central temperature and haemodynamic variables. Left ventricular pressure was measured by a micro-manometer pressure transducer (MPR-500; Millar Instruments, Houston, TX) placed via the right carotid artery. A Vivid 7 scanner (GE Vingmed Ultrasound, Horten, Norway) with 2.5/2.75 MHz probe directly on the heart, was used for echocardiographic and Doppler recordings.

Haemodynamic data regarding systolic and diastolic function obtained from the experiment has been published [3, 14], but is included as background data in the current manuscript. All data presented concerning the electromechanical relations, has been analysed for this study.

### Measurements and analysis

Measurements were obtained at body temperature 38 °C (normal body temperature of the pigs) and 33 °C. 33 °C was chosen representing median temperature recommended from cardiac resuscitation guidelines at that time, with a targeted temperature of 32–34 °C [16]. Measurements were made at both spontaneous heart rate (HR) and during atrial pacing, 100 beats/min (bpm). All measurements and recordings were made in three echocardiographic views over three consecutive heart cycles and the mean values were calculated. The pacing enabled measurements at heart rate 100 bpm, thereby compensating for individual variability and hypothermia induced heart rate reduction. ECG, two-dimensional (2D) and Doppler echocardiography were recorded and analysed offline (EchoPac version 202, GE Healthcare, Horten, Norway).

### Calculations of the electrical events

The ECG lead II was used for electrical measurements. The electrical systole (QT interval), was measured from onset of QRS to end of T wave (Te) in ECG. The QT interval was heart rate corrected according to the Bazett’s formula (QTc) [17]. Te was determined by using the manual tangent method and defined as the intersection of the isoelectric line with the tangent to the steepest downslope of the T wave. Dispersion of repolarization was measured as variation in T peak to T end duration (TpTe). Tp was defined as the first maximum positive or negative deflection of the T wave from the isoelectric line [18]. TpTe was corrected for heart rate using a modified Bazett’s formula (TpTeC = TpTe/√RR).

### Calculation of the mechanical events

**Fig. 1:**
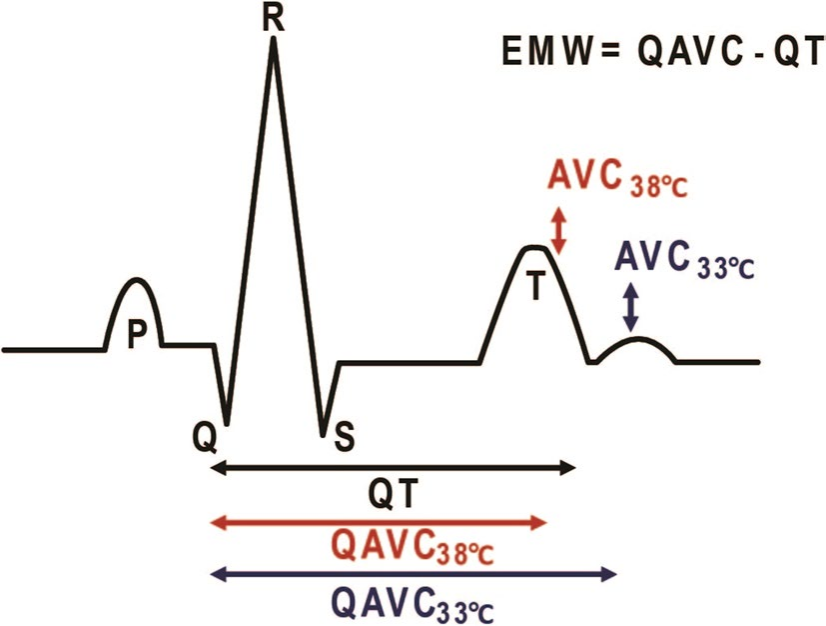
ECG with remarked echocardiographic timing. A schematic drawing of an ECG signal combined with remarked echocardiographic timing of the aortic valve closure (AVC). QT interval is prolonged at 33° schematic drawing of an ECG si Electromechanical window (EMW) is calculated as EMW = QAVC-QT

From echocardiographic apical 4-chamber long axis view, mitral and aortic valve opening and closing were recorded. Isovolumic contraction time (IVCT) and isovolumic relaxation time (IVRT) were measured in long axis view from mitral valve closing (MVC) to aortic valve opening (AVO) and aortic valve closing (AVC) to mitral valve opening (MVO), respectively. Ejection time was measured as the duration of the pulsed wave Doppler signal in left ventricular outflow tract (ET_PW_) and from AVO to AVC. The interval from onset of QRS to the registered AVC represented the duration of the mechanical systole (QAVC). EMW was calculated by subtracting the electrical systole from the duration of the mechanical systole in the same heartbeat, QAVC-QT interval (Fig. 1).

### Global cardiac function

Left ventricular volumes were measured from 2D echocardiographic images (apical 4- and 2-chamber views), and ejection fraction was calculated by the modified Simpson biplane method. Systolic velocity was recorded from mitral ring tissue Doppler velocity images in apical 4-chamber views. Cardiac output was measured by pulmonary artery catheter thermodilution and stroke volume was calculated. Peak systolic left ventricular pressure was recorded by the micro-manometer catheter placed in the left ventricle.

### Regional strain and mechanical dispersion

Longitudinal strain was obtained by speckle tracking echocardiography from the basal and mid segments in three apical left ventricular projections (4-chamber, 2-chamber and long axis, frame rate 63 ± 15 ms). The endocardial border was traced manually, and region of interest was adjusted to fit the myocardial thickness. Segments that failed to track were manually adjusted, and if subsequent failing the segments were excluded. The echocardiographic probe was directly placed on the heart with a subsequent loss of the apical segments, whereof peak strain was derived from 12 segments. Time to peak strain in each segment was defined as the time from Q onset on ECG to peak longitudinal strain in three cycles and averaged. Mechanical dispersion was defined as the standard deviation of time to peak negative strain [19] in the 12 left ventricular segments. Septal and lateral strains were measured from basal segments in 4-chamber view.

### Observer variability

Doppler registered QT and QAVC were re-measured by the same observer to assess intraobserver repeatability. A second observer measured the same variables in seven random selected animals to assess interobserver reproducibility.

### Statistical analyses

Statistical analyses were performed in SPSSv.25 software (SPSS, Inc., Chicago, IL, USA). Parametric data are presented as mean ± standard deviation. Data from normothermia and hypothermia at spontaneous and heart rate 100 bpm were compared by Student’s *t*-test. P-value, *p* < 0.05 was considered statistically significant. Intra- and interobserver variations were analysed by using intraclass correlation coefficient concerning single measures [20].

## Results

All recordings were of good quality. Three animals had a spontaneous heart rate above 100 bpm at 38 °C, and were not paced during normothermia. One animal did not receive pacing due to malfunction of the pacemaker electrode.

### Haemodynamic parameters and myocardial function during hypothermia

During hypothermia mean arterial pressure and left ventricular pressure were reduced at both spontaneous rhythm and heart rate 100 bpm (Table 1). Strain, systolic velocity and cardiac output decreased. Stroke volume and ejection fraction remained unchanged at spontaneous heart rate, but decreased at 100 bpm, indicating that the left ventricle was less tolerant to increased frequency during hypothermia [3, 14].

**Table 1:** Haemodynamic variables

	Spontaneous HR			HR 100 bpm		
	38 °C	33 °C	*p*	38 °C	33 °C	*p*
MAP(mmHg)	62 ± 8	53 ± 9	0.009	65 ± 9	54 ± 7	0.007
SVR(dynes/s/cm^5^)	930 ± 181	966 ± 166	0.591	864 ± 141	915 ± 107	0.325
LVP(mmHg)	84 ± 6	69 ± 11	< 0.001	86 ± 6	67 ± 11	< 0.001
CO(l/min)	4.7 ± 0.9	3.7 ± 0.7	0.001	5.3 ± 0.6	4.0 ± 0.8	< 0.001
SV(ml/beat)	54 ± 7	50 ± 11	0.241	53 ± 7	40 ± 8	< 0.001
EF(%)	57 ± 6	55 ± 8	0.309	60 ± 6	50 ± 5	< 0.001
SS(%)	32 ± 7	23 ± 6	< 0.001	27 ± 7	17 ± 5	< 0.001
SL(%)	30 ± 7	23 ± 7	< 0.001	26 ± 7	17 ± 3	< 0.001
s′(m/s)	0.06 ± 0.01	0.05 ± 0.01	0.023	0.06 ± 0.01	0.05 ± 0.01	0.012

Data are expressed as Mean ± SD

*MAP* mean arterial pressure, *SVR* systemic vascular resistance, *LVP* left ventricular pressure, *CO* cardiac output, *SV* stroke volume, *EF* ejection fraction, *SS* longitudinal septal LV strain, *SL* longitudinal lateral LV strain, *s*′ systolic velocity

### Electromechanical changes at spontaneous heart rate during hypothermia

**Fig. 2:**
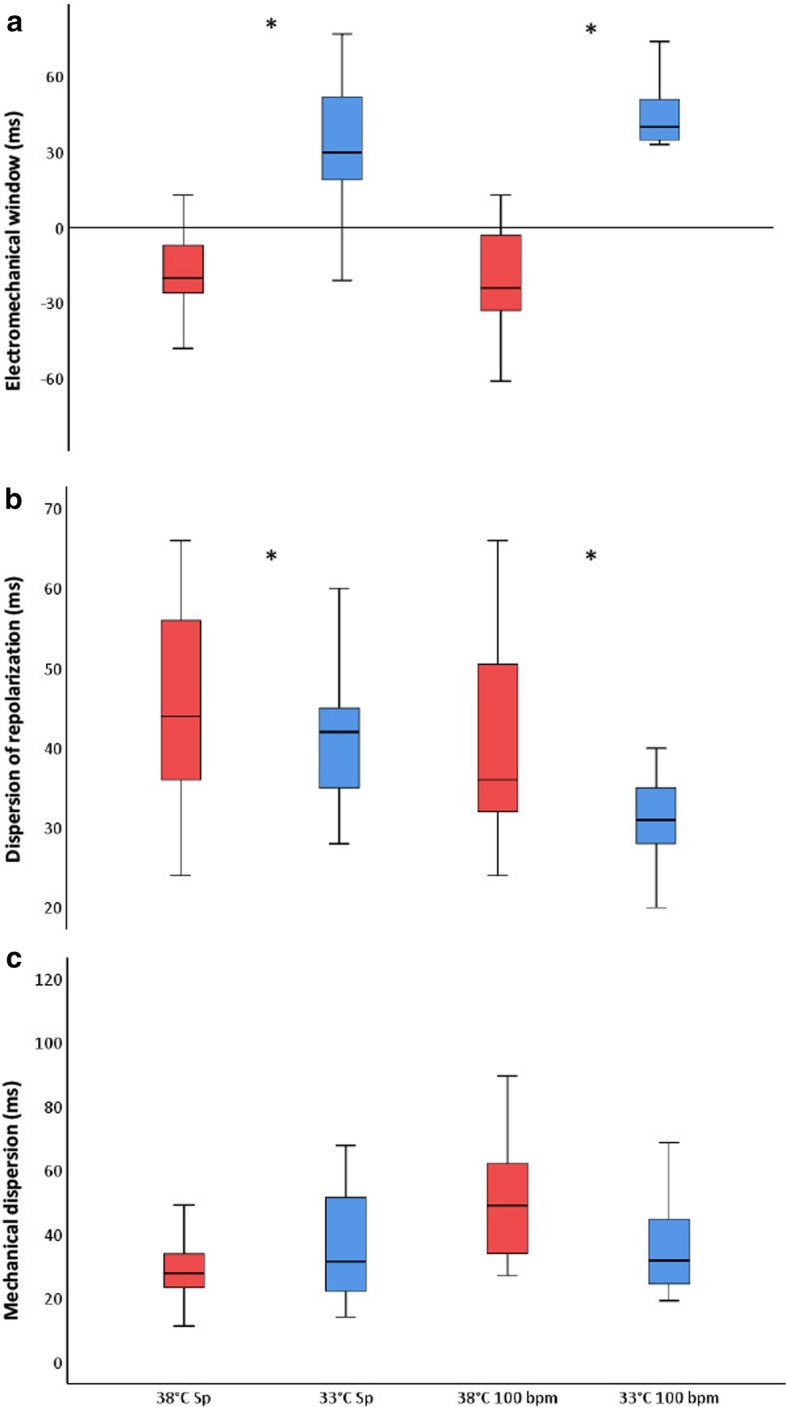
Electromechanical window, dispersion of repolarization and mechanical dispersion. Electromechanical window (**a**), dispersion of repolarization (**b**) and mechanical dispersion (**c**) during normothermia (38 °C) and hypothermia (33 °C) at spontaneous (Sp) and heart rate 100 bpm. The plots are computed from 13 (**a**) and 14 (**b**, **c**) animals at spontaneous heart rate, and 12 (**a**)/13 (**b**, **c**) animals for paced heart rate. Asterisk indicates statistical significance, *p* < 0.05

Hypothermia reduced spontaneous heart rate, prolonged QRS duration and increased QT interval (Table 2). QAVO, IVCT and IVRT did not change. Both the ET_PW_ and the echocardiographic recorded interval from AVO to AVC increased. QAVC increased accordingly, and more than the QT interval (42% vs 20%, respectively). The mechanical systolic duration outlasted the electrical systole, and EMW changed from − 20 ± 19 to 27 ± 34 ms (*p* = 0.001) (Fig. 2). Dispersion of repolarization was reduced also when corrected for heart rate (Table 2). Mechanical dispersion remained unchanged compared to normothermia (Table 3)*.*

**Table 2:** Electrocardiographic variables

	Spontaneous HR			HR 100 bpm		
	38 °C	33 °C	P value	38 °C	33 °C	*p* value
HR(bpm)	88 ± 10	80 ± 7	< 0.001	100 ± 1	100 ± 1	0.326
QRS(ms)	67 ± 10	75 ± 12	< 0.001	64 ± 10	74 ± 11	< 0.001
RR(ms)	694 ± 80	748 ± 69	< 0.001	601 ± 4	602 ± 3	0.177
QT(ms)	395 ± 45	473 ± 51	< 0.001	382 ± 32	406 ± 32	0.005
QTc (ms)	475 ± 50	551 ± 54	< 0.001	494 ± 47	524 ± 43	0.079
TpTe(ms)	45 ± 11	40 ± 10	0.027	41 ± 14	32 ± 7	0.003
TpTeC	56 ± 17	47 ± 12	0.008	55 ± 16	42 ± 12	0.003

Data are expressed as Mean ± SD

*HR* heart rate, *QRS* QRS on ECG, *RR* cardiac cycle interval, *QT* QT interval, *QTc* QT interval corrected for HR, *TpTe* T wave peak to T wave end duration, dispersion of repolarization, *TpTeC* TpTe corrected for heart rate

**Table 3:** Mechanical and electromechanical variables

	Spontaneous HR			HR 100 bpm		
	38 °C	33 °C	*p*	38 °C	33 °C	*p*
QAVO(ms)	91 ± 11	97 ± 10	0.071	89 ± 8	99 ± 7	0.076
QAVC(ms)	353 ± 17	501 ± 24	< 0.001	341 ± 20	456 ± 11	< 0.001
AVO-AVC(ms)	275 ± 47	379 ± 27	< 0.001	257 ± 20	336 ± 17	< 0.001
ET_pw_(ms)	302 ± 36	382 ± 43	0.001	282 ± 22	344 ± 49	0.014
IVCT(ms)	39 ± 11	43 ± 12	0.409	45 ± 2	51 ± 10	0.301
IVRT(ms)	62 ± 13	71 ± 20	0.081	64 ± 19	67 ± 21	0.685
EMW(ms)	− 20 ± 19	27 ± 34	0.001	− 25 ± 26	41 ± 18	< 0.001
MD(ms)	29 ± 11	36 ± 17	0.078	47 ± 16	40 ± 23	0.297

Data are expressed as mean ± SD

*QAVO* Q onset to aortic valve opening, *QAVC* Q onset to aortic valve closing, *AVO-AVC* aortic valve opening to aortic valve closing, *ET*_*pw*_ left ventricular outflow tract ejection time, Doppler registered, *IVCT* isovolumic contraction time, *IVRT* isovolumic relaxation time, *EMW* electromechanical window: QAVC – QT, *MD* mechanical dispersion

### Electromechanical changes at heart rate 100 bpm during hypothermia

At heart rate 100 bpm, electromechanical alterations during hypothermia were similar as during spontaneous heart rate. The QRS complex and the QT interval was widened (Table 2). The mechanical systole was prolonged, but shorter compared to the duration at spontaneous heart rate (Table 3). The QAVC increased with 34% and the QT interval with 6% though the EMW positivity was more pronounced during heart rate 100 bpm, − 25 ± 26 to 41 ± 18 ms, (*p* = 0.001) (Fig. 2)*.* Dispersion of repolarization was reduced (Table 1) and mechanical dispersion unchanged at 100 bpm (Table 3). There were no differences in level of significance for EMW, dispersion of repolarization or mechanical dispersion measured at spontaneous heart rate or at heart rate 100 bpm during hypothermia (Table 4).

**Table 4:** Spontaneous heart rate versus heart rate 100 bpm

	38°Csp	38°Cpm	*p*	33°Csp	33°Cpm	*p*
EMW(ms)	− 20 ± 19	− 25 ± 26	0.281	27 ± 34	41 ± 18	0.099
TpTeC (ms)	56 ± 17	55 ± 16	0.992	47 ± 12	42 ± 12	0.053
MD(ms)	29 ± 11	47 ± 16	< 0.001	36 ± 17	40 ± 23	0.632

Data are expressed as mean ± SD

*sp* spontaneous heart rate, *pm* atrial pacing; 100 bpm, *EMW* electromechanical window: QAVC – QT, *TpTeC* dispersion of repolarization, corrected for heart rate, *MD* mechanical dispersion

### Observer variability

Intraobserver correlation coefficient for QT and QAVC were 0.99 (95% confidence interval (CI) 0.98–1.0) and 0.99 (95% CI 0.99–1.0). Interobserver correlation coefficient for the same parameters were 0.99 (95% CI 0.98–1.0) and 0.99 (95% CI 0.77–1.0).

## Discussion

In this experimental study, targeted hypothermia increased both electrical and mechanical systolic duration. Indeed, the mechanical systole outlasted the electrical systole independently of heart rate and therefore the EMW became positive. Dispersion of repolarization was slightly reduced, and mechanical dispersion was unchanged, at both spontaneous and at heart rate 100 bpm.

### The shift in electromechanical window

Cardiac arrhythmic diseases are primarily perceived as electrical disorders. Prolonged QT interval with EMW negativity is an independent pro-arrhythmic predictor. This arrhythmogenicity may be a consequence not only of electrical alterations, but likely also of mechanical influences. In one study increased EMW negativity for any QTc in long QT syndrome was found, indicating an electrical-independent mechanical influence [6]. In our study, we found that despite the increased QT interval there was significant change to EMW positivity during hypothermia, due to the even more prolonged mechanical systolic duration. The QRS interval and IVCT were slightly prolonged, but gave only a small contribution to the extended QAVC at spontaneous heart rate. The increase in AVO to AVC interval was the main contributor to the prolonged mechanical systolic duration. These results support the theory that electromechanical alterations during targeted hypothermia may be consequences of mechanical independent influences as well.

In our in vivo model, baseline EMW was slightly negative at both heart rates. The normal value for EMW in land race pigs during normothermia is unknown, but an open chest model can lead to a prolonged QT interval which may contribute to EMW negativity [21]. In an ex vivo model, isolated Langendorff-perfused Göttingen minipig hearts baseline EMW was 134 ms [22]. However, an ex vivo model may have substantial cardiac electromechanical influence [23]. During hypothermia we found that EMW changed from negative to positive. A corresponding tendency with preserved or increased EMW during cooling is previously reported [24, 25].

### Electrical dispersion

TpTe is a marker for dispersion of repolarization with a strong association to arrhythmic risk. Whether the ECG measured dispersion represents transmural repolarization, global or both is still not clarified [26, 27]. In our model, single lead ECG from lead II was used for TpTe measurements. We found no increase, but rather a reduction in dispersion of repolarization during hypothermia. Previously published experimental and clinical results have shown no significant increase in transmural dispersion of repolarization during moderate hypothermia despite increased action potential duration and slowing of conduction velocity [28, 29]. Inducing repolarization abnormalities by ventricular pacing did neither increase risk of arrhythmia at 33 °C compared to normothermia and pro-arrhythmic parameters such as slowing of conduction velocity and prolonged action potential duration were attenuated at 33 °C [30]. Experimental studies actually indicate a direct anti arrhythmic effect on the myocardial cells by increased membrane stability during targeted hypothermia [5, 31]. However, at lower temperatures, experimental studies with targeted temperature have shown increased dispersion of repolarization [28, 32]. Our findings are in accordance with comparable experimental data. The reported slightly reduced dispersion of repolarization at 33 °C in our study may be a consequence of the registered lead II which represents global repolarization with less correlation to transmural dispersion.

### Mechanical dispersion

The prolongation and slowing of systolic contraction during targeted hypothermia at 33 °C is previously described [3, 14]. However, it is not known whether this is due to asynchronous activation and relaxation of left ventricular segments with a prolonged systole, or as synchronous slowed prolongation of all segments. Pathological mechanical dispersion occurs as a mechanical consequence of electrical alterations or myocardial dysfunction [33] and correlates with increased QT interval, contraction duration and EMW negativity [34, 35].

Overall, we found no increase in mechanical dispersion but slightly prolonged ventricular depolarization, prolonged QT interval with reduced dispersion of repolarization and increased ventricular contraction duration. The registered increase in contraction duration may be explained by a synchronous contraction prolongation since there was no increase in dispersion of repolarization. However, it is still somewhat unclear how electrical and mechanical activities are coupled. The absence of mechanical dispersion does not necessarily indicate a synchronous pattern. Mechanical dispersion is measured as SD of time to peak systolic strain. Contraction end is not taken into account, which may be displaced. Hypothermia induced electrical delay with increased action potentials or affected electrical conduction in the myocardium, might have an additional coexisting influence on the systolic contraction pattern. Based on our experiments it is difficult to draw any conclusion regarding either mechanism.

### Clinical implications

The beneficial and harmful effects of targeted hypothermia and the optimal temperature are not completely clarified. After cardiac arrest it might be difficult to distinguish whether myocardial dysfunction, arrhythmias or other adverse events are due to myocardial injury or to hypothermia itself. This may contribute to a possible restrain regarding clinical implementation [36, 37]. Our experimental model describes changes in the electromechanical relations during hypothermia in healthy animal hearts with a clinical translational value but confined by the experimental setting with anatomical and physical differences and unexposed pathology compared to patients with cardiac arrest. Our findings in this experimental model indicate an electromechanical change that could infer decreased arrhythmia susceptibility during hypothermia at 33 °C but the isolated effect from targeted hypothermia on electromechanical relations in healthy pig hearts should be interpreted cautiously. However, this study makes an important contribution to further explore the effect of hypothermia on the electromechanical relations. Clinical studies are required to clarify whether these electromechanical alterations during hypothermia are accommodating in humans.

## Limitations

Three-lead ECG were connected to the echocardiographic scanner. TpTe interval measurements from the bipolar limb lead II have limitations as discussed above. ECG TpTe interval from bipolar limb leads is thought to represent global dispersion, including apicobasal and interventricular dispersion of repolarization. Transmural dispersion of repolarization is most represented in precordial leads and is suggested to be the preferred leads when estimating arrhythmic risk [26]. Our study protocol was not designed to evaluate arrhythmic risk, but to study electromechanical relations. Digital ECG and automatic analyses with multi-lead representations may have made the results more valid, but were not available due to the already accomplished animal study. The animals in our model represented their own control with no other adjustment than temperature and HR during the experiments. The methods used to identify electrical intervals were identical for all measurements and the reproducibility of the measurements was good.

The open chest model may have altered the preload and afterload conditions [21]. However, we analysed the absolute change in the parameters during stable phases. The pigs were anaesthetized with isoflurane and morphine after induction with pentobarbital. This is routinely applied and recommended in experimental settings when cardiothoracic surgery is needed [23], despite the well-known pharmacological impact on the cardiovascular system and the autonomic balance [38–40]. The anaesthetics were maintained at the same rate with no modifications throughout the experiment. In addition, the possible pharmacological heart rate effect was superseded by the temperature effect, and compensated by atrial paced heart rate 100 bpm during normo- and hypothermia.

## Conclusion

Targeted hypothermia increased the duration of both the electrical and mechanical systole. The prolonged duration of the mechanical systole was relatively greater and outlasted the electrical systole at hypothermia. This led to a positive EMW. Hypothermia slightly reduced dispersion of repolarization, while mechanical dispersion remained unchanged. Further clinical studies are required to elucidate whether these electromechanical relations during hypothermia are presented in humans.

## Data Availability

The datasets used and analysed during the current study are available from the corresponding author on reasonable request.

## Abbreviations

ECG: Electrocardiogram
QT interval: Electrical systole
QTc: Corrected QT interval
bpm: Beats per minute
2D: Two dimensional
EMW: Electromechanical window
TpTe: T wave peak to T wave end; electrical dispersion of repolarization
TpTeC: Corrected TpTe interval
AVO: Aortic valve opening
AVC: Aortic valve closing
ET_pw_: Ejection time by pulsed wave Doppler signal in left ventricular outflow tract
QAVC: Q onset to aortic valve closing; mechanical systole
MAP: Mean arterial pressure
SVR: Systemic vascular resistance
LVP: Left ventricular pressure
CO: Cardiac output
SV: Stroke volume
EF: Ejection fraction
SS: Strain septal
SL: Strain longitudinal
*s*′: Mitral ring systolic velocity
HR: Heart rate
QRS: QRS complex on ECG
RR: Interval from R to R between two QRS complexes
IVCT: Isovolumic contraction time
IVRT: Isovolumic relaxation time
MD: Mechanical dispersion
